# Índice de Inflamação Imune Sistêmica é Preditor de Eventos Cardiovasculares Adversos Maiores em Pacientes com Infarto Agudo do Miocárdio com Supradesnivelamento do Segmento ST

**DOI:** 10.36660/abc.20210412

**Published:** 2022-04-25

**Authors:** Faysal Saylik, Tayyar Akbulut

**Affiliations:** 1 Van Education and Research Hospital Department of Cardiology Van Turquia Van Education and Research Hospital - Department of Cardiology, Van – Turquia

**Keywords:** Infarto do Miocárdio, Cardiopatias Congênitas, Vasos Coronários

## Abstract

**Fundamento:**

O índice de inflamação imune sistêmica (SII, systemic immune-inflammation index) tem sido descrito como um novo marcador prognóstico em tumores e doenças cardiovasculares.

**Objetivos:**

Investigar a associação entre eventos cardiovasculares adversos em pacientes com infarto agudo do miocárdio com supradesnivelamento do segmento ST (IAMCSST).

**Métodos:**

Foi realizado um estudo observacional retrospectivo em 843 pacientes com IAMCSST. Os pacientes foram divididos em dois grupos segundo valores medianos de SII. A análise de regressão de Cox foi usada para detectar preditores independentes de eventos adversos cardiovasculares. A melhora na capacidade discriminatória pela adição do SII aos fatores de risco tradicionais – idade, hipertensão, diabetes mellitus, e sexo masculino para eventos adversos maiores foi calculada por estatística c, melhora da discriminação integrada (IDI), e melhora na reclassificação. Um valor de p bilateral <0,05 foi considerado estatisticamente significativo.

**Resultados:**

O grupo com SII elevado apresentou idade mais avançada que o grupo com SII baixo (61,2±11,2 e 59,2±7,9, respectivamente, p=0,002). O grupo com SII elevado apresentou taxas mais altas de morte cardiovascular, infarto do miocárdio não fatal, acidente vascular cerebral não fatal, hospitalização por insuficiência cardíaca, revascularização, e eventos cardiovasculares adversos maiores que no grupo com SII baixo. O SII foi um preditor independente de todos os eventos mencionados. A adição do SII aos fatores de risco tradicionais melhorou sua capacidade discriminatória para eventos cardiovasculares. O SII foi superior à razão neutrófilo-linfócito e à razão plaqueta-linfócito para predizer eventos adversos cardiovasculares.

**Conclusão:**

O SII foi um preditor independente de eventos adversos maiores em pacientes com IAMCSST e pode ser usado para melhorar a predição de eventos adversos risco, especialmente se combinado com fatores de risco tradicionais.

## Introdução

A aterosclerose é a principal causa de doença cardiovascular, e a principal causa de morte em todo o mundo.^1^ A presença de inflamação na área aterosclerótica tem um papel crítico na formação e ruptura da placa.^2^ A formação de placa aterosclerótica vulnerável e de trombo, que resulta na interrupção de fluxo sanguíneo coronário, é o mecanismo fisiopatológico primário em pacientes com infarto agudo do miocárdio com supradesnivelamento do segmento ST (IAMCSST).^3^ O tratamento de escolha de paciente com IAMCSST é a intervenção coronária percutânea (ICP) primária. Apesar dos avanços no tratamento antitrombótico e nas técnicas de reperfusão, pacientes com IAMCSST ainda têm um prognóstico ruim.

A estratificação de risco precoce de pacientes em alto risco de eventos cardiovasculares adversos futuros é crucial. Estudos prévios mostraram que a inflamação e a trombose foram associadas ao início, progressão, e prognóstico do IAMCSST.^4^ Assim, a descoberta de novos biomarcadores inflamatórios tornou-se um tópico de interesse para detectar pacientes de alto risco e fornecer informações sobre o prognóstico.^5 , 6^ As plaquetas e os leucócitos exercem papeis importantes no desenvolvimento de aterosclerose e síndromes coronárias agudas. Contagens plaquetárias mais elevadas podem refletir processos inflamatórios destrutivos e estado pró-trombótico.^7^ Os neutrófilos sãos os primeiros leucócitos a migrar do sangue para o miocárdio lesionado, e contagens de neutrófilos altas foram associadas a uma maior área de infarto, complicações mecânicas, e mortalidade.^8 , 9^ Por outro lado, os linfócitos controlam a resposta imune, promovendo menor dano do miocárdio.^10^ O índice de inflamação imune sistêmica (SII, do inglês *systemic immune-inflammation index* ) é um marcador simples, determinado com base nas contagens de neutrófilos, plaquetas, e linfócitos [SII= (neutrófilo x plaqueta) / linfócito] para determinar o estado imune e inflamatório. Recentemente, o SII foi considerado um preditor independente do prognóstico em várias doenças, incluindo tumores e doenças cardiovasculares.^1 , 11 , 12^ Nosso objetivo foi investigar a capacidade preditiva do SII para desfechos clínicos adversos em pacientes com IAMCSST após ICP primária.

## Materiais e métodos

Um total de 1187 pacientes consecutivos admitidos em nosso hospital com IAMCSST, submetidos à ICP primária entre 2012 e 2020 foram incluídos retrospectivamente no estudo. Desses, foram excluídos 334 pacientes com revascularização coronária prévia, doença hematológica, oncológica, ou inflamatória, infecção ativa, insuficiência hepática ou renal, doença cardíaca valvular grave, e choque cardiogênico na admissão. Ainda, pacientes com dados faltantes e pacientes cujos dados de seguimento não puderam ser obtidos não foram incluídos. Assim, 843 pacientes participaram do estudo. O estudo foi conduzido de acordo com a Declaração de Helsinki de 1975, revisada em 2008, e aprovado pelo comitê de ética local.

### Definições

O diagnóstico de STEMI foi estabelecido com base das diretrizes atualizadas da definição universal de infarto do miocárdio (IM).^13 , 14^ As características basais, histórias clínicas, dados laboratoriais, e imagens angiográficas dos pacientes foram obtidos do banco de dados do hospital. Todas as amostras sanguíneas dos pacientes foram obtidas na admissão ao departamento de emergência. As análises foram realizadas usando equipamentos de análises hematológicas Beckman Coulter LH 780 (Beckman Coulter, FL, EUA), e parâmetros bioquímicos analisados em um aparelho Roche Cobas 6000 c501 (Roche, Mannheim, Germany). O SII foi calculado usando a fórmula SII= (P x N) / L, onde P = contagem total de plaquetas no sangue periférico; N = contagem de neutrófilos; e L = contagem de linfócitos. O clearance de creatinina foi calculado usando a equação de Cockcroft- Gault: clearance de creatinina = [140 – idade em anos x peso (kg)] / (72 x creatinina sérica [mg/dL]) para homens e foi corrigido multiplicando por 0,85 para mulheres. Hipertensão (HT) foi definida como pressão arterial sistólica ≥ 140 mm Hg e/ou pressão arterial diastólica ≥ 90 mmHg em duas ou mais medidas, ou uso atual de drogas anti-hipertensivas. O diagnóstico de diabetes mellitus (DM) foi dado com base na glicemia de jejum ≥ 126 mg/dL ou glicemia pós-prandial ≥ 200 mg/dL, ou uso de medicamentos antidiabéticos. Foi considerado tabagista o paciente que fumou durante o mínimo de seis meses contínuos no ano anterior. História familiar de doença arterial coronariana (DAC) foi definida como história de DAC em parentes de primeiro grau com idade inferior a 55 anos para mulheres e 65 anos para homens.

Foi realizada angiografia coronariana padrão transradial ou transfremoral, à critério do técnico responsável, usando a técnica de Seldinger. Os pacientes receberam 300 mg de acetilsalicílico, dose de ataque de inibidor de P_2_Y_12_ (clopidogrel), e uma dose padrão de heparina não fracionada (70-100 U/Kg) antes do procedimento. O uso de bloqueadores de receptor de glicoproteína IIb/IIIa (Tirofiban) foi realizado a critério do operador. Os angiogramas foram analisados por dois investigadores experientes, cegos a todos os dados clínicos. Fluxo TIMI (trombólise no infarto do miocárdio, ou do inglês *thrombolysis in myocardial infarction* ) e o grau de perfusão miocárdica TIMI (TIMI *myocardial perfusion grade* , TMPG) foram avaliados conforme definido anteriormente.^15 - 17^ A ausência de refluxo foi definida como TIMI 0, I e II no angiograma final. A embolização distal foi determinada como um defeito de preenchimento distal novo de um ou mais ramos periféricos da artéria coronária da artéria relacionada ao infarto, com uma oclusão abrupta distal ao local da intervenção coronária.

### Acompanhamento

Dados clínicos do acompanhamento foram reunidos a partir do banco de dados do hospital e da farmácia, ou por contato telefônico com pacientes e/ou parentes. Registros hospitalares ou certificados de óbito foram usados para determinar a causa de morte.

#### Desfechos

O desfecho composto primário foram eventos adversos cardiovasculares maiores (MACE), que é uma combinação de morte cardiovascular, IM não fatal, e acidente vascular cerebral (AVC) não fatal. Mortes por IM, arritmias fatais, parada cardíaca, e mortes atribuídas à insuficiência cardíaca ou a outras condições cardíacas foram classificadas como morte cardiovascular. IM não fatal foi definida como recorrência de dor torácica e/ou alteração no segmento ST no eletrocardiograma, com uma nova elevação dinâmica nos níveis de troponina I e CKMB (aumento > 20% do basal). AVC não fatal foi caracterizado como um bloqueio em um dos vasos que leva sangue para o cérebro, evidenciado por ressonância magnética ou tomografia computadorizada (TC), e um déficit neurológico recente com duração superior a 24 horas.

#### Análises estatísticas

Todas as análises estatísticas foram realizadas usando o programa SAS (SAS/STAT, *University Edition* , SAS Institute Inc, NC, EUA). Uma vez que existiam mais de um desfecho e diferentes ponto de corte, os pacientes foram divididos em dois grupos – pacientes com SII alto (>554,9) e pacientes com SII baixo (<554,9), com base na mediana do SII. A normalidade dos dados foi avaliada pelo teste de Kolmogorov-Smirnov. Variáveis contínuas com distribuição normal foram apresentadas em média (intervalo interquartil). Variáveis categóricas foram expressas em números (porcentagens). O teste t de Student ou teste U de Mann-Whitney foi usado para comparar variáveis contínuas entre os grupos, conforme apropriado. O teste do qui-quadrado de Pearson ou o teste exato de Fisher foi usado para comparação das variáveis categóricas. O modelo de riscos proporcionais de Cox, com cálculo da razão de risco (HR, *hazard ratio* ) foi usado para detectar preditores de eventos adversos em pacientes com IAMCSST test. Incluímos variáveis aos modelos de acordo com os tamanhos dos eventos na análise multivariada de Cox para evitar a superestimação. Para avaliar a melhoria na capacidade discriminatória de ventos adversos em longo prazo no modelo basal (com os fatores de risco tradicionais – idade, sexo masculino, DM, e HT) com a adição do SII, usamos a estatística de concordância de Harrell com teste de DeLong,^18^ calculamos a melhora da discriminação integrada (IDI, *integrated discrimination improvement* ), e melhora na reclassificação (NRI, *net reclassification improvement* .^19^ Realizamos a análise da curva Característica de Operação do Receptor (ROC, do inglês, *receiver operating characteristic curve* ) para determinar o ponto de corte ótimo do SII pelo índice de Youden, e cálculo da área sob a curva (AUC). O Critério de Informação de Akaike (AIC),^20^ o Critério de Informação Bayesiano (BIC),^21^ o logaritmo da probabilidade (-2LL), e NagelkarkeR^2^ foram usados para avaliar as comparações das capacidades das variáveis – razão neutrófilo-linfócito (RNL), razão plaqueta-linfócito (RPL), e SII para predizer MACE. Níveis mais baixos de AIC, BIC, e -2LL, e níveis mais altos de NagelkarkeR^2^ indicam um melhor ajuste do modelo.^22^ A diferença nas taxas de sobrevida livre de eventos entre os grupos segundo SII foi analisada usando a curva de Kaplan-Meier, e o teste de log-rank usado para avaliar significância estatística. Um valor de p<0,05 foi considerado estatisticamente significativo em todos os testes.

## Resultados

Características basais, dados laboratoriais e angiográficos dos 843 pacientes dos grupos SII baixo e SII alto estão descritos na Tabela 1 . O grupo com SII alto apresentou idade mais avançada que o grupo com SII baixo (p=0,002). A presença de DAC familiar foi maior no grupo com SII alto que no grupo com SII baixo (p=0,004). Contagem total de leucócitos, contagem de plaquetas, contagem de neutrófilos, e níveis de LDL colesterol foram mais altos no grupo com SII alto, enquanto a contagem de linfócitos foi mais baixa. Quanto às características angiográficas, observou-se uma maior frequência de presença de mais de dois *stents* implantados, doença de múltiplos vasos, embolização distal, e ausência de refluxo no grupo com SII alto em comparação ao grupo com SII baixo. TMPG e fluxo TIMI foram piores no grupo SII alto que no grupo SII baixo. O grupo SII alto apresentou maiores taxas de angioplastia coronária transluminal percutânea (ACTP) (p=0,002), e taxas mais baixas de implante direto (sem pré-dilatação) de *stents* (p=0,008) que o grupo com SII baixo.

**Tabela 1:** – Características basais e angiográficas da população estudada segundo o índice de inflamação imune sistêmica (SII)

Variáveis	SII<554,9 (N=421)	SII≥554,9 (N=422)	Valor p
Idade, anos	59,2(7,9)	61,2(11,2)	0,002
Sexo masculino, n (%)	277(65,8)	288(68,3)	0,449
Diabetes, n (%)	90(21,4)	111(26,3)	0,093
Hipertensão, n (%)	131(31,1)	148(35,1)	0,222
Tabagismo, n (%)	129(30,6)	156(36,9)	0,052
Hiperlipidemia, n (%)	154(36,6)	171(40,5)	0,239
História familiar de DAC, n (%)	74(17,6)	108(25,6)	**0,005**
IMC, Kg/m^2^	23,8(22-25,3)	23,7(21,4-26,6)	0,589
**Medicação prévia**			
AAS, n (%)	98(23,3)	123(29,2)	0,053
iECA/BRA, n (%)	154(36,6)	176(41,7)	0,127
Betabloqueadores, n (%)	147(34,9)	165(39,1)	0,209
Diuréticos, n (%)	37(8,8)	52(12,3)	0,096
Estatinas, n (%)	75(17,8)	94(22,3)	0,106
FEVE, %	42,3(7)	41,6(10,5)	0,256
CLT, 10^3^ mL	7,6(6-8,9)	7,9(6,4-9,7)	**0,007**
Hemoglobina, mg/dL	14,2(1,1)	14,2(1,7)	0,651
Plaquetas, /mm^3^	204,1(173,6-228,7)	243,7(189-279)	**<0,0001**
Neutrófilos, 10^3^ mL	6,3(5,4-7)	6,5(5,4-7,8)	**0,004**
Linfócitos, 10^3^/mL	2,7(2-3,3)	2,1(1,3-3,4)	**<0,0001**
Creatinina sérica, mg/dL	0,9(0,2)	0,9(0,3)	0,825
Colesterol total, mg/dL	171,3(147,1-191,6)	163,4(129,8-204,6)	0,120
LDL colesterol, mg/dL	111,9(102,8-121,5)	117,5(99,7-135,9)	**0,006**
HDL colesterol, mg/dL	43(36,3-48,5)	40,4(31,5-51,8)	0,057
Triglicerídeos, mg/dL	137,2(98,6-177,7)	129,8(87,9-204,1)	0,858
Glicose, mg/dL	116(29,5)	114,5(37,6)	0,534
**Angiografia**			
Tempo entre dor e dilatação (balão), h	4,3(2,8-5,5)	4,4(2,5-6,5)	0,152
Número total de stents > II	27(6,4)	82(19,4)	**<0,0001**
Doença de múltiplos vasos, n (%)	86(20,4)	123(29,2)	**0,0034**
Comprimento total do stent, mm	23,7(4,1)	23,9(6)	0,598
**Procedimento, n (%)**			**0,0008**
Implante direto (sem pré-dilatação)	135(32,1)	101(23,9)	0,008
ACTP + stent	274(65,1)	289(68,5)	NS
Somente ACTP	12(2,8)	32(7,6)	0,002
TMPG>II, n (%)	272(64,6)	228(54)	**0,0018**
Fluxo TIMI pós-procedimento >III, n (%)	410(97,4)	374(89,6)	**<0,0001**
Uso de inibidor GpIIb/IIIa, n (%)	41(9,7)	77(18,3)	**0,0004**
Embolização distal, n (%)	2(0,5)	15(3,6)	**0,002**
Sem refluxo, n (%)	11(2,6)	48(11,4)	**<0,0001**
Interrupção da TAPD <30 dias	6(1,4)	11(2,6)	0,224
Interrupção da TAPD <6 meses	22(5,3)	29(6,9)	0,316
Adesão à TAPD por 12 meses	399(94,8)	393(93,1)	0,317

*DAC: doença arterial coronariana, IMC: índice de massa corporal, DAC: doença arterial coronariana, AAS: ácido acetilsalicílico, iECA: inibidores de enzima conversora de angiotensina, BRA: bloqueador de receptor de angiotensina, FEVE: fração de ejeção do ventrículo esquerdo, FEVE: fração de ejeção do ventrículo esquerdo, CTL: contagem total de leucócitos, LDL: lipoproteína de baixa densidade, HDL: lipoproteína de alta densidade, h: horas, ACTP: angioplastia coronária transluminal percutânea, TIMI: trombólise no infarto do miocárdio, TMPG: grau de perfusão miocárdica TIMI, TAPD: terapia antiplaquetária dupla; valores expressos em média (desvio padrão), mediana (intervalo interquartil) ou n (%).*

### Desfechos clínicos

**Figura 1:**
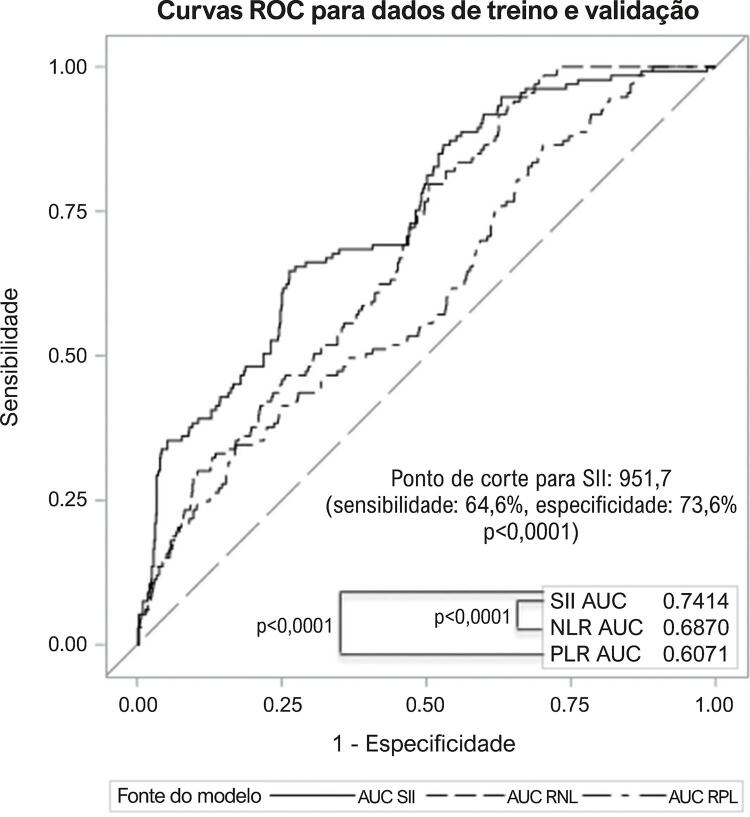
– Comparação de Curvas ROC (Características de Operação do Receptor ou receiver operating characteristic) de índice de inflamação imune sistêmica (SII), razão neutrófilo-linfócito (RNL) e razão plaqueta-linfócito (RPL) para eventos cardiovasculares maiores (MACE) em pacientes com infarto agudo do miocárdio com supradesnivelamento do segmento ST (IAMCSST); AUC: área sob a curva.

**Figura 2:**
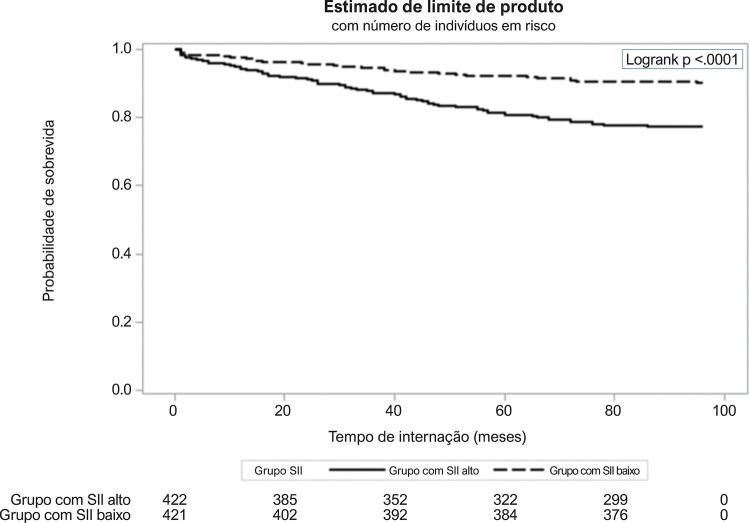
– Curvas de Kaplan-Meier dos grupos com índice de inflamação imune sistêmica (SII) alto e SII baixo quanto a eventos cardiovasculares maiores (MACE).

O período mediano de acompanhamento foi de 34,2 meses (IIQ: 8,6 – 63,9). Os eventos adversos clínicos foram comparados entre os grupos com SII alto e SII baixo ( Tabela 2 ). No seguimento, morte cardíaca, IM não fatal, AVC não fatal, hospitalização por insuficiência cardíaca congestiva (ICC), revascularização, e frequência de MACE foram maiores no grupo com SII alto. Resultados da análise de regressão de Cox são apresentados na Tabela 2 . SII elevado foi associado a um risco 3,6 vezes maior de morte cardíaca, risco 2,79 vezes maior de IM não fatal; risco 2,98 vezes maior no AVC não fatal; risco 11,1 vezes maior de internação por ICC, risco 4,11 vezes maior de revascularização (ICP ou cirurgia de bypass da artéria coronária, CABG), e a um risco 8,52 vezes maior de MACE. Na análise da curva ROC, o ponto de corte de 951,7 para SII apresentou uma sensibilidade de 64,6% e especificidade de 73,6% para discriminação de MACE (AUC = 0,741, p<0,0001). Na comparação ROC, o SII apresentou uma melhor capacidade discriminatória para MACE em comparação a RNL (p<0,0001) e RPL (p<0,0001) ( Figura 1 ). Comparações de desempenho diagnóstico entre RNL, RPL e SII mostraram que o SII apresentou melhor capacidade preditiva para MACE que RNL e RPL ( Tabela 3 ). A curva de sobrevida Kaplan Meier mostrou maior ocorrência de MACE no grupo com SII elevado que no grupo com baixo SII ( Figura 2 ).

**Tabela 2:** – Desfechos clínicos dos pacientes com infarto agudo do miocárdio com supradesnivelamento do segmento ST (IAMCSST) estratificados por índice de inflamação imune sistêmica (SII) e análise de regressão de Cox

Desfechos clínicos	SII< 554,9 N=421	SII≥554,9 N=422	Valor p	Regressão de Cox HR (IC95%)	Valor p
Morte cardíaca	17(4)	46(10,9)	0,0002	3,064(1,754-5,353)	<0,0001^a^
Infarto do miocárdio não fatal	20(4,8)	54(12,8)	<0,0001	2,787(1,658-4,684)	0,0001^b^
AVC não fatal	6(1,4)	16(3,8)	0,0312	2,984(1,163-7,654)	0,023^c^
Hospitalização por ICC	15(3,6)	70(16,6)	<0,0001	11,114(4,137-29,858)	<0,0001^d^
Revascularização (ICP ou CABG)	57(13,5)	94(22,3)	0,0009	4,113(1,887-8,966)	0,0004^e^
MACE	41(9,7)	92(21,8)	<0,0001	8,516(4,458-16,268)	<0,0001^e^

*a Ajustado por idade, sexo, hipertensão, diabetes mellitus, lipoproteína de baixa densidade (LDL) colesterol b Ajustado por idade, sexo, hipertensão, diabetes mellitus, LDL colesterol, história familiar de doença arterial coronariana c Ajustado por idade d Ajustado por idade, hipertensão, diabetes mellitus, LDL colesterol, sexo, história familiar de doença arterial coronariana, EF (fração de ejeção) e Ajustado por idade, hipertensão, diabetes mellitus, LDL colesterol, sexo, história familiar de doença arterial coronariana, EF (fração de ejeção), índice de massa corporal, creatinina, glicose HR: hazard ratio; ICC: insuficiência cardíaca congestiva; ICP: intervenção coronária percutânea; CABG: bypass da artéria coronária (coronary artery by-pass graft); MACE: eventos cardiovasculares maiores (major adverse cardiovascular events); AVC: acidente vascular cerebral*

**Tabela 3:** – Comparação do desempenho diagnóstico dos preditores para eventos adversos cardiovasculares maiores

Variáveis	-2LL	AIC	BIC	Nagelkarke R^2^
SII	665,1	669,1	678,6	0,1367
RNL	707,6	711,6	721,1	0,0551
RPL	713,4	717,4	726,9	0,0434

*LL: logaritmo da probabilidade; AIC: Critério de Informação de Akaike; BIC: Critério de Informação Bayesiano; RNL: razão neutrófilo-linfócito; RPL: razão plaqueta-linfócito.*

### Valor preditivo adicional do SII

A adição do SII ao modelo basal com fatores de risco tradicionais (idade, DM, HT, e sexo masculino) melhorou a predição de morte cardíaca, IM não fatal, AVC não fatal, internação por ICC, revascularização, e MACE, conforme demonstrado pela estatística C ( Tabela 4 ). A melhora na capacidade discriminatória pela adição do SII também foi confirmada pelo IDI de 0,0857, com melhora de 49% na pelo aumento significativo no NRI para morte cardíaca, IM não fatal (NRI:0,4936, IDI:0,0743), AVC não fatal (NRI:0,4655, IDI:0,0307), hospitalização por ICC (NRI:0,7183, IDI:0,1448), revascularização (NRI:0,2971, IDI:0,0231), e MACE (NRI:0,4539, IDI:0,1073) ( Tabela 4 ), sugerindo que a adição de SII pode melhorar significativamente a capacidade de predição de eventos adversos em comparação ao uso de somente fatores de risco tradicionais em pacientes com IAMCSST.

**Tabela 4:** – Avaliação de modelos preditivos para eventos adversos cardíacos**

	Estatística C (IC95%)*	NRI (IC95%)	IDI (IC95%)
**Morte cardíaca**			
Fatores de risco tradicionais	0,704(0,633-0,776)	Ref	Ref
Fatores de risco tradicionais +SII	0,780(0,713-0,847)	0,4962(0,2661,0,7264)	0,0857(0,058,0,1133)
Valor p	0,02	0,0002	<0,0001
**Infarto do miocárdio não fatal**			
Fatores de risco tradicionais	0,641(0,571-0,710)	Ref	Ref
Fatores de risco tradicionais +SII	0,757(0,688-0,826)	0,4936(0,2772,0,7101)	0,0743(0,054,0,0946)
Valor p	0,0006	<0,0001	<0,0001
**AVC não fatal**			
Fatores de risco tradicionais	0,615(0,481-0,750)	Ref	Ref
Fatores de risco tradicionais +SII	0,756(0,631-0,881)	0,4655(0,0871,0,844)	0,0307(0,0158,0,0457)
Valor p	0,043	0,031	<0,0001
**Hospitalização por ICC**			
Fatores de risco tradicionais	0,884(0,852-0,914)	Ref	Ref
Fatores de risco tradicionais +SII	0,939(0,918-0,961)	0,7183(0,5413,0,8953)	0,1448(0,1031,0,1865)
Valor p	<0,0001	<0,0001	<0,0001
**Revascularização (ICP ou CABG)**			
Fatores de risco tradicionais	0,923(0,904-0,942)	Ref	Ref
Fatores de risco tradicionais +SII	0,931(0,915-0,949)	0,2971(0,1254,0,4687)	0,0231(0,0089,0,0371)
Valor p	0,036	0,0009	0,0014
**MACE**			
Fatores de risco tradicionais	0,644(0,592-0,696)	Ref	Ref
Fatores de risco tradicionais +SII	0,754(0,703-0,804)	0,4539(0,2806,0,6271)	0,1073(0,0834,0,1311)
Valor p	<0,0001	<0,0001	<0,0001

IDI: melhora da discriminação integrada (integrated discrimination improvement); NRI: melhora na reclassificação (net reclassification improvement); SII: índice de inflamação imune sistêmica; ICC: insuficiência cardíaca congestiva; ICP: intervenção coronária percutânea; CABG: bypass coronário (coronary artery by-pass graft); MACE: eventos cardiovasculares maiores. * Valores p para estatística c: teste DeLong. ** Fatores de risco tradicionais: idade, hipertensão, diabetes mellitus, e sexo masculino. AVC: acidente vascular cerebral.

## Discussão

Este estudo mostrou que pacientes com valores de SII elevados apresentaram maior frequência de morte cardíaca, AVC não fatal, hospitalização por ICC, revascularização, e MACE que pacientes com baixos valores de SII. Ainda, SII foi um preditor independente desses desfechos adversos. A adição do SII a fatores de risco tradicionais, tais como idade, HT, DM, e sexo masculino, melhorou a capacidade de predição de eventos cardiovasculares adversos em pacientes com STEMI após ICP primária. Finalmente, o SII foi superior a outros biomarcadores convencionais tais como RNL e RPL na predição de MACE.

O IM é causado pela formação de trombos nas artérias coronárias resultante da ruptura de placas coronárias ou erosão da placa de ateroma.^3^ O processo inflamatório e a trombose exercem papeis importantes na iniciação e progressão dessa condição.^23^ Os neutrófilos emitem armadilhas extracelulares ( *neutrophil extracellular traps* , NETs), que foram detectadas nas placas de ateroma e possivelmente têm um papel na formação de placa de aterosclerose e aumento na estabilidade do trombo.^24^ Zhang et al.^25^ relataram que a contagem de neutrófilos associou-se independentemente com MACE em pacientes com STEMI.^25^ Por outro lado, os linfócitos refletem um processo inflamatório calmo e regulado que suprime a resposta imune e menos dano no miocárdio.^26^ Uma contagem mais baixa de linfócitos foi associada a um maior risco de doença cardiovascular e mortalidade.^27^ Quando ativadas, as plaquetas liberam quantidades consideráveis de quimiocinas pró-inflamatórias e citocinas de grânulos alfa, o que leva ao estado imune destrutivo e pró-trombótico. Estudos prévios mostraram uma associação entre contagem de plaquetas e MACE.^7 , 28^ Biomarcadores derivados desses três tipos celulares foram amplamente investigados e apresentados na literatura como marcadores prognósticos dado ao menor custo e facilidade de obtenção e cálculo. Ainda, estudos com pacientes com IAMCSST têm relatado que tanto a RNL como a RPL são fortes preditores independentes de MACE.^6 , 29^

Recentemente, o SII surgiu como um marcador potencial, com base nas células inflamatórias, incluindo neutrófilos, linfócitos, e plaquetas, e tem sido associado a piores desfechos e várias condições.^1 , 11 , 12^ Gok et al.^30^ relataram que o SII associou-se com embolia pulmonar maciça aguda e se mostrou superior a outros índices relacionados à inflamação, semelhante aos nossos resultados no presente estudo. Um estudo prévio de Erdoğan et al.^31^ mostrou uma associação significativa entre SII e gravidade da DAC. O SII foi associado com desfechos ruins no pós-operatório após cirurgia de *bypass* coronário sem circulação extracorpórea.^12^ Agus et al.^32^ relataram que o SII relacionou-se independentemente com mortalidade hospitalar em pacientes com endocardite infecciosa. Além disso, o SII associou-se com desfechos clínicos adversos em pacientes idosos (65-85 anos de idade) com síndrome coronária aguda.^33^ Embora esse estudo^33^ tenha apresentado resultados similares aos nossos, em nosso estudo, incluímos pacientes adultos de todas as idades, e apresentando somente IAMCSST. Outro estudo conduzido por Yang et al.^1^ propôs que o SII foi um preditor independente de eventos adversos em pacientes com DAC, incluindo pacientes com angina estável, IAMCSST e IAM sem supradesnivelamento do segmento ST.^1^ Em estudos recentes, o SII apresentou melhor valor prognóstico que a RNL e a RPL.^34^ Para evitar interação e multicolinearidade, nós não incluímos RNL ou RPL nos modelos de regressão com SII. Contudo, de acordo com os estudos mencionados acima, a AUC calculada da análise ROC e as comparações dos ajustes dos modelos incluindo -2LL, AIC, BIC e NagelkarkeR^2^demonstraram que o SII pode apresentar um melhor ajuste que a NLR e a RPL para estratificação de risco de pacientes com IAMCSST submetidos à ICP primária.

Uma vez que a predição precoce de eventos adversos em pacientes com IAMCSST em alto risco, submetidos à ICP primária, é crucial para as estratégias de tratamento e acompanhamento, um SII elevado pode exercer um papel na classificação de risco e tratamento desses pacientes.

As principais limitações do presente estudo foram o tamanho relativamente pequeno da amostra e seu delineamento retrospectivo e unicêntrico. Além disso, coletamos dados de um período de oito anos, a partir de registros hospitalares e, por isso, é possível que tenha ocorrido viés de seleção devido a variáveis de confusão que não foram medidas (afetando eventos adversos), e exclusão de pacientes com variáveis faltantes. As contagens de plaquetas, neutrófilos, e linfócitos foram registradas somente uma vez, na admissão. Medidas durante internação ou acompanhamento não foram registradas, e o impacto das mudanças dessas variáveis sobre eventos cardiovasculares é ainda incerto. Ensaios grandes, randomizados controlados, poderiam fornecer evidências mais claras sobre a capacidade preditiva do SII para eventos adversos em pacientes com IAMCSST.

## Conclusão

Neste estudo, um SII elevado apresentou uma reação independente com eventos cardiovasculares adversos, incluindo morte cardíaca, IM não fatal, AVC não fatal, hospitalização por insuficiência cardíaca, revascularização, e MACE compostos em pacientes com IAMCSST após ICP primária. Além disso, a predição de risco de MACE melhorou com a adição do SII aos fatores de risco tradicionais. SII foi superior à RNL e à RPL na predição de eventos adversos em pacientes com IAMCSST após ICP primária. O SII é um preditor facilmente calculável que poderia ser usado na detecção de pacientes com IAMCSST em alto risco, submetidos à ICP primária.
