# Supplementary material for: Índice de Inflamação Imune Sistêmica é Preditor de Eventos Cardiovasculares Adversos Maiores em Pacientes com Infarto Agudo do Miocárdio com Supradesnivelamento do Segmento ST
Source: Arq Bras Cardiol. 2022 Apr 25;119(1):14–22. [Article in Portuguese] doi: 10.36660/abc.20210412 (PMC9352114; doi:10.36660/abc.20210412)
Supplement: Translation

# Translation: Systemic Immune-Inflammation Index Predicts Major Cardiovascular Adverse Events in Patients with ST-Segment Elevated Myocardial Infarction

**Authors:** Faysal Saylik, Tayyar Akbulut

**Affiliations:** 1 Turkey Van Education and Research Hospital - Department of Cardiology, Van – Turkey

**Keywords:** Myocardial Infarction, Heart Defects, Congenital, Coronary Vessels

## Abstract

**Background:**

The systemic immune-inflammation index (SII) has been reported as a new prognostic marker in tumors and cardiovascular diseases

**Objective:**

To investigate the association of SII with adverse cardiovascular events in patients with ST-segment elevated myocardial infarction (STEMI).

**Methods:**

A retrospective observational study was conducted on 843 patients with STEMI. Patients were divided into two groups based on the median value of SII. Major adverse cardiovascular events were compared between SII groups. Cox regression analysis was used for detecting independent predictors of cardiovascular adverse events. The improvement of discrimination ability by adding SII to the traditional risk factors such as age, hypertension, diabetes mellitus, and male gender for major adverse events was calculated by c-statistics, integrated discrimination improvement, and net reclassification improvement. A two-sided p-value <0.05 was considered significant.

**Results:**

High SII group was older than the low SII group (61.2±11.2, 59.2±7.9, respectively, p=0.002). The high SII group had higher rates of cardiac death, nonfatal myocardial infarction, nonfatal stroke, hospitalization for heart failure, revascularization, and composite major adverse cardiovascular events than the low SII group. SII was an independent predictor of all events mentioned above. Adding SII to traditional risk factors improved their discrimination ability for cardiovascular events. SII was superior to the neutrophil-to-lymphocyte and platelet-to- lymphocyte ratios for predicting cardiovascular adverse events.

**Conclusion:**

SII was an independent predictor of major adverse events in patients with STEMI and may be used to improve the prediction of adverse events, especially when combined with traditional risk factors.

## Introduction

Atherosclerosis is the leading cause of cardiovascular disease, and continues to be the leading cause of death worldwide.^1^ The presence of inflammation in the atherosclerotic area has a critical pathophysiological role for plaque formation and rupture.^2^ Vulnerable atherosclerotic plaque and thrombus formation that results in the cessation of coronary blood flow is the primary pathophysiologic mechanism in patients with ST-segment elevation myocardial infarction (STEMI).^3^ The first choice of treatment for STEMI patients is primary percutaneous coronary intervention (pPCI). Despite advances in antithrombotic treatment and reperfusion techniques, patients with STEMI still have a poor prognosis.

Early risk stratification of patients who are at high risk for future adverse cardiovascular events is very crucial. Previous studies have shown that inflammation and thrombosis have been linked to the initiation, progression, and prognosis of STEMI.^4^ So, the discovery of novel inflammatory biomarkers has be of interest to detect high-risk patients and to provide information for prognosis.^5 , 6^ Platelets and leukocytes play crucial roles in the development of atherosclerosis and acute coronary syndromes. Higher platelet counts might reflect destructive inflammatory processes and prothrombotic status.^7^ Neutrophils are the first leukocytes to migrate from the blood to the damaged myocardial area, and increased neutrophil counts have been associated with large infarct size, mechanical complications, and mortality.^8 , 9^ In contrast, lymphocytes control the immune response, providing less myocardial damage.^10^ The systemic immune-inflammation index (SII) is a simple marker, which has been established based on the neutrophil, platelet, and lymphocyte counts [SII= (neutrophil x platelet) / lymphocyte] to determine the inflammatory and immune status. Recently, SII was considered an independent predictor of prognosis in several conditions, including tumors and cardiovascular diseases.^1 , 11 , 12^ We aimed to investigate the predictive ability of SII for adverse clinical outcomes in patients with STEMI after pPCI.

## Materials and Methods

A total of 1,187 consecutive patients admitted to our hospital with STEMI who underwent pPCI between 2012 and 2020 were retrospectively included in this study. Of them, 344 patients with previous coronary revascularization, hematological, oncological, or inflammatory disease, active infection, hepatic or renal insufficiency, severe valvular heart disease, and cardiogenic shock at admission were excluded. Also, patients with missing data and patients whose follow-up data could not be obtained were not included in the study population. Finally, the study was completed with 843 patients. The study was carried out according to the Declaration of Helsinki of 1975, as revised in 2008 and approved by the local ethics committee.

## Definitions

The diagnosis of STEMI was made based on the updated guidelines for the universal definition of myocardial infarction (MI).^13 , 14^ Baseline characteristics, clinical histories, laboratory measurements, and angiographic images of patients were obtained from the hospital database. All blood samples of patients were obtained at admission to the emergency department. Blood measurements were analyzed using a Beckman Coulter LH 780 hematology analyzer (Beckman Coulter, FL, USA) for hematologic parameters and a Roche Cobas 6000 c501 (Roche, Mannheim, Germany) for biochemical parameters. The SII was calculated with the formula SII= (P x N) / L, where P = total peripheral platelet count; N = neutrophil count (N), and L = lymphocyte count. Creatinine clearance was calculated using the Cockcroft- Gault equation: Creatinine clearance = ([140 - age in years] x weight[kg]) / (72 x serum creatinine [mg/dL]) for men and was corrected by multiplying with 0.85 for women. Hypertension (HT) was diagnosed as systolic blood pressure 140 mm Hg and/or diastolic blood pressure ≥ 90 mm Hg at least two times or the use of current antihypertensive drugs. Diabetes mellitus (DM) was diagnosed based on fasting glucose ≥ 126 mg/dL or postprandial glucose ≥ 200 mg/dL or the use of antidiabetic drugs. Cigarette smoking was defined as patients who had smoked for at least six months continuously during the past year. A family history of coronary artery disease (CAD) was defined as a history of CAD in first-degree relatives less than 55 years for women and 65 years for men.

## Angiographic definitions

At the operator’s discretion, the standard coronary angiography (CAG) was performed through the transradial or transfemoral approach using the Seldinger technique. Acetylsalicylic acid (300 mg), a loading dose of P_2_Y_12_inhibitors (Clopidogrel), and a standard dose of unfractionated heparin (70-100 U/kg) were given to all patients before the CAG procedure. The use of glycoprotein IIb/IIIa receptor blockers (tirofiban) was left to the operator’s discretion. The angiographic images of patients were carefully reviewed by two experienced investigators who were blinded to all clinical data. Thrombolysis in myocardial infarction (TIMI) flow and TIMI myocardial perfusion grade (TMPG) were assessed as previously defined.^15 - 17^ No-reflow was defined TIMI 0, I, and II in the final angiogram. Distal embolization was determined as a new distal filling defect of one or more peripheral coronary artery branches of the infarct-related artery, with an abrupt occlusion distal to the coronary intervention site.

### Follow-up

Clinical follow-up data were gathered from the hospital and pharmacy database or through telephone calls with the patients and/or their relatives. Hospital records or death certificates were used to determine the cause of death.

#### Endpoints

The primary composite endpoint was major cardiovascular adverse events (MACE), which is a combination of cardiovascular death, nonfatal MI, and nonfatal ischemic stroke. Deaths due to MI, life-threatening arrhythmias, cardiac arrest, and deaths attributed to heart failure or other cardiac conditions were all classified as cardiovascular death. Non-fatal MI was defined as the recurrence of chest pain and/or new electrocardiographic ST-segment change with a new dynamic elevation in troponin I and CKMB levels (>20% increase from baseline). Nonfatal ischemic stroke was characterized as a blockage in a blood vessel supplying blood to the brain, as evidenced by magnetic resonance imaging (MRI) or computed tomography (CT) scans, and a recent neurologic deficit that lasted for more than 24 hours.

#### Statistical Analyses

All statistical analyses were carried out on SAS University Edition (SAS/STAT, SAS Institute Inc, NC, USA). Because there were more than one endpoint and different cut-off points, patients were divided into two groups as high (≥554.9) and low (<554.9) SII based on median SII value. The normality of data was tested using the Kolmogorov-Smirnov test. Continuous variables with a normal distribution were expressed as mean (standard deviation), while those without a normal distribution were presented as median (interquartile range), and categorical variables were expressed as numbers (percentages). The independent Student’s t-test or Mann-Whitney U test was used for comparing continuous variables between groups as appropriate. The Pearson Chi-square test or Fisher exact test was used for comparison of categorical variables. Hazard ratios (HR) for Cox proportional hazards regression, adjusted with covariates were used to detect predictors of adverse events in patients with STEMI. We included variables into models according to the event sizes in multivariable Cox regression analysis to avoid overestimation. To assess the improvement in discrimination ability for long-term adverse events of the baseline model (with traditional risk factors – age, male gender, DM, and HT), with the addition of SII, the Harrell’s concordance statistics (c-statistics) with DeLong test,^18^ integrated discrimination improvement (IDI), and net reclassification improvement (NRI) were calculated.^19^ The receiver operating characteristic (ROC) curve analysis was performed to determine the optimal cutoff value of the SII using the Youden index, and the area under the curve (AUC) was obtained. The Akaike information criterion (AIC),^20^ the Bayesian information criterion (BIC),^21^ -2 Log likelihood (-2LL), and NagelkarkeR^2^were used to assess the comparisons of the abilities of variables – neutrophil-to-lymphocyte (NLR), platelet-to-lymphocyte ratio (PLR), and SII – to predict MACE. Lower levels of AIC, BIC, and -2LL and higher levels of NagelkarkeR^2^indicate a better model fit.^22^ The difference in event-free survival rates between SII groups was analyzed using the Kaplan-Meier survival curve, and the log-rank test was used to evaluate the statistical significance. A p-value of <.05 was considered significant in all statistical analyses.

## Results

Baseline characteristics, laboratory data, and angiographic features of 843 patients of the high and low SII groups are described in Table 1 . The high SII group was older than the low SII group (p=0.002). The presence of familial CAD was more frequent in the high SII group than in the low SII group (p=0.005). White blood cell (WBC) count, platelet count, neutrophil count, and LDL cholesterol levels were more elevated in the high SII group, whereas lymphocyte count was lower. Regarding angiographic data, there was a higher frequency of implanted stents > II, multivessel disease, distal embolization, and no-reflow in the high SII group compared to the low SII group. TMPG and TIMI flow were worse in the high SII group than in the low SII group. The high SII group had higher rates of percutaneous transluminal coronary angioplasty (PTCA) (p=0.002) and lower rates of direct stenting (p=0.008) rates than the low SII.

**Table 1:** – Baseline and angiographic characteristics of the study population by SII*

Variables	SII<554.9 (N=421)	SII≥554.9 (N=422)	p value
Age, years	59.2(7.9)	61.2(11.2)	0.002
Male gender, n (%)	277(65.8)	288(68.3)	0.449
Diabetes, n (%)	90(21.4)	111(26.3)	0.093
Hypertension, n (%)	131(31.1)	148(35.1)	0.222
Smoking, n (%)	129(30.6)	156(36.9)	0.052
Hyperlipidemia, n (%)	154(36.6)	171(40.5)	0.239
Family history of CAD, n (%)	74(17.6)	108(25.6)	**0.005**
BMI, kg/m^2^	23.8(22-25.3)	23.7(21.4-26.6)	0.589
**Previous Medication**			
ASA, n (%)	98(23.3)	123(29.2)	0.053
ACEi/ARB, n (%)	154(36.6)	176(41.7)	0.127
Beta blocker, n (%)	147(34.9)	165(39.1)	0.209
Diuretic, n (%)	37(8.8)	52(12.3)	0.096
Statin, n (%)	75(17.8)	94(22.3)	0.106
LVEF, %	42.3(7)	41.6(10.5)	0.256
WBC, 10^3^mL	7.6(6-8.9)	7.9(6.4-9.7)	**0.007**
Haemoglobin, mg/dL	14.2(1.1)	14.2(1.7)	0.651
Platelet, /mm^3^	204.1(173.6-228.7)	243.7(189-279)	**<0.0001**
Neutrophil, 10^3^mL	6.3(5.4-7)	6.5(5.4-7.8)	**0.004**
Lymphocyte, 10^3^/mL	2.7(2-3.3)	2.1(1.3-3.4)	**<0.0001**
Serum Creatinine, mg/dL	0.9(0.2)	0.9(0.3)	0.825
Total cholesterol, mg/dL	171.3(147.1-191.6)	163.4(129.8-204.6)	0.120
LDL cholesterol, mg/dL	111.9(102.8-121.5)	117.5(99.7-135.9)	**0.006**
HDL cholesterol, mg/dL	43(36.3-48.5)	40.4(31.5-51.8)	0.057
Triglyceride, mg/dL	137.2(98.6-177.7)	129.8(87.9-204.1)	0.858
Glucose, mg/dL	116(29.5)	114.5(37.6)	0.534
**Angiographic Characteristics**			
Pain to balloon time, hours	4.3(2.8-5.5)	4.4(2.5-6.5)	0.152
Total number of stents > II	27(6.4)	82(19.4)	**<0.0001**
Multivessel disease, n (%)	86(20.4)	123(29.2)	**0.0034**
Total stent length, mm	23.7(4.1)	23.9(6)	0.598
**Procedure, n (%)**			**0.0008**
Direct stenting	135(32.1)	101(23.9)	0.008
PTCA+stenting	274(65.1)	289(68.5)	NS
Only PTCA	12(2.8)	32(7.6)	0.002
TMPG>II, n (%)	272(64.6)	228(54)	**0.0018**
Postprocedural TIMI flow >III, n (%)	410(97.4)	374(89.6)	**<0.0001**
Use of GpIIb/IIIa inhibitor, n (%)	41(9.7)	77(18.3)	**0.0004**
Distal embolization, n (%)	2(0.5)	15(3.6)	**0.002**
No reflow, n (%)	11(2.6)	48(11.4)	**<0.0001**
DAPT interruption <30 days	6(1.4)	11(2.6)	0.224
DAPT interruption <6 months	22(5.3)	29(6.9)	0.316
Adherence for DAPT for 12 months	399(94.8)	393(93.1)	0.317

*SII: systemic immune-inflammation index, CAD: coronary artery disease, BMI: body mass index, ASA: acetylsalicylic acid, ACEi: angiotensin converting enzyme inhibitors, ARB: angiotensin receptor blocker, LVEF: left ventricular ejection fraction, WBC: White blood cell, LDL: low density cholesterol, HDL: high density cholesterol, PTCA: percutaneous transluminal coronary angioplasty, TIMI: thrombolysis in myocardial infarction, TMPG: TIMI myocardial perfusion grade, DAPT: dual antiplatelet therapy; values shown as mean (standard deviation), median (interquartile range), n (%).*

### Clinical outcomes

The median follow-up was 34.2 months (IQR: 8.6- 63.9). The clinical adverse events were compared between the high and the low SII groups ( Table 2 ). In the follow-up, cardiac death, nonfatal MI, nonfatal stroke, hospitalization for congestive heart failure (CHF), revascularization, and frequency of MACE were higher in the high SII group. Results of the Cox regression analysis results are shown in Table 2 . High SII was linked to a 3.06-fold increased risk of cardiac death, 2.79-fold increased risk of nonfatal MI, 2.98-fold-increased risk of nonfatal stroke, 11.1-fold increased risk of hospitalization for CHF, 4.11-fold increased risk of revascularization (PCI or coronary artery by-pass graft [CABG]), and 8.52-fold increased risk of MACE. In ROC analysis, a cutoff value of 951.7 for SII had 64.6% sensitivity and 73.6% specificity for discrimination of MACE (AUC=0.741, p<0.0001). In ROC comparison, SII had a better discrimination ability for MACE than NLR and PLR (p<0.0001 for both, Figure 1 ). Diagnostic performance comparisons between NLR, PLR, and SII showed that SII had a higher prediction ability for MACE than NLR and PLR ( Table 3 ). The Kaplan-Meier survival curve showed that the high SII group had a higher occurrence of MACE compared to the low SII group ( Figure 2 ).

**Table 2:** – Clinical outcomes in ST-segment elevated myocardial infarction (STEMI) patients stratified by systemic-immmune inflammation index (SII) and Cox regression analysis

Clinical Outcomes	SII< 554.9 N=421	SII≥554.9 N=422	p value	Cox regression analysis HR (IC95%)	p value
Cardiac death	17(4)	46(10.9)	0.0002	3.064(1.754-5.353)	<0.0001^a^
Nonfatal myocardial infarction	20(4.8)	54(12.8)	<0.0001	2.787(1.658-4.684)	0.0001^b^
Nonfatal stroke	6(1.4)	16(3.8)	0.0312	2.984(1.163-7.654)	0.023^c^
Hospitalization for CHF	15(3.6)	70(16.6)	<0.0001	11.114(4.137-29.858)	<0.0001^d^
Revascularization (PCI or CABG)	57(13.5)	94(22.3)	0.0009	4.113(1.887-8.966)	0.0004^e^
MACE	41(9.7)	92(21.8)	<0.0001	8.516(4.458-16.268)	<0.0001^e^

*a Adjusted for age, gender, hypertension, diabetes mellitus, low-density lipoprotein (LDL) cholesterol b Adjusted for age, gender, hypertension, diabetes mellitus, LDL cholesterol, family of coronary artery disease c Adjusted for age d Adjusted for age, hypertension, diabetes mellitus, LDL cholesterol, gender, family coronary artery disease (CAD),EF (ejection fraction) e Adjusted for age, hypertension, diabetes mellitus, LDL cholesterol, gender, family of CAD, EF (ejection fraction), body mass index, creatinine, glucose HR: hazard ratio; CHF: congestive heart failure; PCI: percutaneous coronary intervention; CABG: coronary artery by-pass graft; MACE: major adverse cardiovascular events.*

**Figure 1:** – Comparisons of Receiver-operating characteristics (ROC) curves of systemic immune-inflammation index (SII), neutrophil-to-lymphocyte ratio (NLR), and platelet-to-lymphocyte ratio (PLR) for major adverse cardiovascular events (MACE) in patients with ST-segment elevated myocardial infarction.

**Table 3:** – Comparison of diagnostic performance of predictors for major adverse cardiovascular events

Variables	-2LL	AIC	BIC	Nagelkarke R^2^
SII	665.1	669.1	678.6	0.1367
NLR	707.6	711.6	721.1	0.0551
PLR	713.4	717.4	726.9	0.0434

*LL: log likelihood, AIC: akaike criterion index, BIC: bayesian criterion index, SII: systemic immune-inflammation index, NLR: neutrophil-to-lymphocyte ratio, PLR: platelet-to-lymphocyte ratio.*

**Figure 2:** – Kaplan-Meier survival curves of high and low SII groups for MACE. SII: Systemic immune-inflammation index; MACE: Major adverse cardiovascular events.

### Additional predictive value of SII

Adding SII to the baseline model with traditional risk factors (age, DM, HT, and male gender) improved the prediction of cardiac death, nonfatal MI, nonfatal stroke, hospitalization for CHF, revascularization, and MACE, as demonstrated by the significant increase in the C-statistics ( Table 4 ). Discrimination improvement by adding SII was also confirmed by an IDI of 0.0857, with 49% improvement in NRI for cardiac death, nonfatal MI (NRI:0.4936, IDI:0.0743), nonfatal stroke (NRI:0.4655, IDI:0.0307), hospitalization for CHF (NRI:0.7183, IDI:0.1448), revascularization (NRI:0.2971, IDI:0.0231), and MACE (NRI:0.4539, IDI:0.1073) ( Table 4 ), suggesting that adding SII may provide a significantly better prediction of adverse events than traditional risk factors alone in patients with STEMI.

**Table 4:** – Evaluation of predictive models for cardiac adverse events**

	C-statistics (95% CI)*	NRI (95% CI)	IDI (95% CI)
**Cardiac death**			
Traditional risk factors	0.704(0.633-0.776)	Ref	Ref
Traditional risk factors +SII	0.780(0.713-0.847)	0.4962(0.2661.0.7264)	0.0857(0.058.0.1133)
p value	0.02	0.0002	<0.0001
**Nonfatal myocardial infarction**			
Traditional risk factors	0.641(0.571-0.710)	Ref	Ref
Traditional risk factors +SII	0.757(0.688-0.826)	0.4936(0.2772.0.7101)	0.0743(0.054.0.0946)
p value	0.0006	<0.0001	<0.0001
**Nonfatal stroke**			
Traditional risk factors	0.615(0.481-0.750)	Ref	Ref
Traditional risk factors +SII	0.756(0.631-0.881)	0.4655(0.0871.0.844)	0.0307(0.0158.0.0457)
p value	0.043	0.031	<0.0001
**Hospitalization for CHF**			
Traditional risk factors	0.884(0.852-0.914)	Ref	Ref
Traditional risk factors +SII	0.939(0.918-0.961)	0.7183(0.5413.0.8953)	0.1448(0.1031.0.1865)
p value	<0.0001	<0.0001	<0.0001
**Revascularization (PCI or CABG)**			
Traditional risk factors	0.923(0.904-0.942)	Ref	Ref
Traditional risk factors +SII	0.931(0.915-0.949)	0.2971(0.1254.0.4687)	0.0231(0.0089.0.0371)
p value	0.036	0.0009	0.0014
**MACE**			
Traditional risk factors	0.644(0.592-0.696)	Ref	Ref
Traditional risk factors +SII	0.754(0.703-0.804)	0.4539(0.2806.0.6271)	0.1073(0.0834.0.1311)
p value	<0.0001	<0.0001	<0.0001

NRI: net reclassification improvement, IDI: integrated discrimination improvement, SII: systemic immune-inflammation index, CHF: congestive heart failure, PCI: percutaneous coronary intervention, CABG: coronary artery by-pass graft, MACE: major adverse cardiovascular events. * p values for c-statistics: DeLong test. ** Traditional risk factors: age, hypertension, diabetes mellitus, and male gender.

## Discussion

This study has shown that patients with high SII values had higher frequencies of cardiac death, nonfatal MI, nonfatal stroke, hospitalization for CHF, revascularization, and MACE than patients with low SII values. Furthermore, SII was an independent predictor of these adverse outcomes. Adding SII to traditional risk factors such as age, HT, DM, and male gender improved the prediction ability for adverse cardiovascular events in STEMI patients after pPCI. Finally, SII was superior to other conventional biomarkers such as NLR and PLR in predicting MACE.

MI is caused by thrombus formation in the coronary arteries as a result of coronary plaque rupture or erosion of the atheromatous plaque.^3^ The inflammatory process and thrombosis were found to play significant roles in the initiation and progression of this condition.^23^ Neutrophils release neutrophil extracellular traps (NETs), which have been detected in atheromatous plaque and might play a causative role in atherosclerotic plaque formation and increased thrombus stability.^24^ Zhang et al.^25^ found that neutrophil counts were independently associated with MACE in STEMI patients.^25^ In contrast, lymphocytes reflect a calm and regulated inflammatory process that causes suppressed immune response and less myocardial damage.^26^ Lower lymphocyte counts have been linked to a higher risk of cardiovascular disease and mortality.^27^ Upon activation, platelets release considerable quantities of proinflammatory chemokines and cytokines from alfa granules, which lead to destructive immune and prothrombotic status. Previous studies have shown that platelet count was associated with MACE.^7 , 28^ Biomarkers derived from these three cell types mentioned above were widely researched and reported as prognostic markers in the literature due to their relatively low cost and easiness for acquisition and calculation. Also, studies with STEMI patients have reported that both NLR and PLR are useful and powerful independent predictors of MACE.^6 , 29^

Recently, SII has been emerged as a potential marker based on inflammatory cells, including neutrophils, lymphocytes, and platelets, and has been reported to be associated with worse outcomes in several conditions.^1 , 11 , 12^ Gok et al.^30^ reported that SII was associated with massive acute pulmonary embolism and was superior to other inflammation-based indexes, similarly to what we observed in the present study. A previous study by Erdogan et al.^31^ showed a significant association between SII and CAD severity. SII was found to be associated with poor postoperative outcomes after elective off-pump coronary artery bypass surgery.^12^ Agus et al.^32^ reported that SII was independently related to in-hospital mortality in patients with infective endocarditis. In addition, SII has been associated with adverse clinical outcomes in acute coronary syndrome in patients aged between 65 and 85.^33^ Although this study^33^ had similar results to ours, in our study, we included adult patients of all ages, and with STEMI only. Another study conducted by Yang et al.^1^ proposed that SII was an independent predictor of adverse events in CAD patients, including stable angina pectoris, non-STEMI, and STEMI patients.^1^ In recent studies, the prognostic value of SII was reported to be better than PLR and NLR.^34^ To avoid multicollinearity and interaction, we did not put NLR and PLR in the Cox regression models with SII. However, in concordance with the abovementioned studies, AUC calculated from ROC analysis and model fit comparisons including -2LL, AIC, BIC, and NagelkarkeR^2^ demonstrated that SII might fit better than NLR and PLR for risk stratification of STEMI patients undergoing pPCI.

Because the early prediction of adverse events in high-risk patients with STEMI undergoing pPCI is crucial for treatment and follow-up strategies, high SII might serve a role in the risk classification and treatment assignment for these patients.

The relatively small sample size and retrospective and single-center design were the major limitations of this study. In addition, we collected data from an eight-year period, from hospital records, so there might be selection bias due to unmeasured confounding variables affecting adverse events and exclusion of patients with missing variables. The platelet, neutrophil, and lymphocyte counts were recorded only once upon admission. The in-hospital or follow-up measurements were not recorded, and the impacts of changes of these variables on adverse cardiovascular events remained uncertain. Large randomized controlled trials could provide more definitive evidence about the predictive ability of SII for clinical adverse events in patients with STEMI.

## Conclusion

This study identified that high SII was independently related to adverse cardiovascular events, including cardiac death, nonfatal MI, nonfatal stroke, hospitalization for heart failure, revascularization, and composite MACE in patients with STEMI after pPCI. Furthermore, the risk prediction of MACE was improved by adding SII to traditional risk factors. SII was superior to NLR and PLR in the prediction of adverse events in STEMI patients after pPCI. Finally, SII is an easily calculable predictor that could be used to detect high-risk patients with STEMI undergoing pPCI.
